# Seroprevalence and molecular characterization of viral hepatitis and HIV co-infection in the Central African Republic

**DOI:** 10.1371/journal.pone.0291155

**Published:** 2024-05-09

**Authors:** Parvine Basimane-Bisimwa, Giscard Wilfried Koyaweda, Edgarthe Ngaïganam, Ulrich Vickos, Ornella Anne Demi Sibiro, Brice Martial Yambiyo, Benjamin Seydou Sombié, Pulchérie Pélembi, Sandrine Moussa, Claudine Bekondi, Tamara Giles-Vernick, Alexandre Manirakiza, Muriel Vray, Narcisse Patrice Joseph Komas

**Affiliations:** 1 Institut Pasteur de Bangui, Viral Hepatitis Laboratory, Bangui, Central African Republic; 2 Université Evangélique en Afrique (UEA), Bukavu, Democratic Republic of Congo; 3 International Center Advanced for Research and Training (ICART), Bukavu, Democratic Republic of Congo; 4 Hôpital Général de Référence de Panzi, Bukavu, Democratic Republic of Congo; 5 Laboratory of Arbovirus, Haemorrhagic Fevers, Institut Pasteur de Bangui, Emerging Virus and Zoonosis, Bangui, Central African Republic; 6 Institut Pasteur de Bangui, Epidemiology Service, Bangui, Central African Republic; 7 Centre National de Recherche et de Formation sur le Paludisme, Ouagadougou, Burkina Faso; 8 Institut Pasteur de Bangui, Service des Retrovirus-VIH, Bangui, Central African Republic; 9 Institut Pasteur de Bangui, Centre de Ressources Biologiques, Bangui, Central African Republic; 10 Institut Pasteur-Université Paris Cité, Anthropology & Ecology of Disease Emergence Unit, INSERM, Paris, France; 11 Institut Pasteur-Université Paris Cité, Unit of Epidemiology of Emergent Infections, INSERM, Paris, France; CEA, FRANCE

## Abstract

**Background:**

The Central African Republic (CAR) is one of the countries with the highest prevalence of viral hepatitis infection in the world. Coinfection with HIV increases the morbidity and mortality beyond that of mono-infection with either hepatitis or HIV. The present study describes the geographic distribution of viral hepatitis infections and molecular characterization of these viruses in the CAR.

**Methodology:**

Out of 12,599 persons enrolled during the fourth Multiple Indicator Cluster Survey of 2010 in the CAR, 10,621 Dried Blood Spot (DBS) samples were obtained and stored at -20°C. Of these DBS, 4,317 samples were randomly selected to represent all regions of the CAR. Serological tests for hepatitis B, D, and C viruses were performed using the ELISA technique. Molecular characterization was performed to identify strains.

**Results:**

Of the 4,317 samples included, 53.2% were from men and 46.8% from women. The HBsAg prevalence among participants was 12.9% and that HBc-Ab was 19.7%. The overall prevalence of HCV was 0.6%. Co-infection of HIV/HBV was 1.1% and that of HBV/HDV was 16.6%. A total of 77 HBV, 6 HIV, and 6 HDV strains were successfully sequenced, with 72 HBV (93.5%) strains belonging to genotype E and 5 (6.5%) strains belonging to genotype D. The 6 HDV strains all belonged to clade 1, while 4 recombinants subtype were identified among the 6 strains of HIV.

**Conclusion:**

Our study found a high prevalence of HBV, HBV/HDV and HBV/HIV co-infection, but a low prevalence of HCV. CAR remains an area of high HBV endemicity. This study’s data and analyses would be useful for establishing an integrated viral hepatitis and HIV surveillance program in the CAR.

## Introduction

Hepatitis is defined as an inflammation of the liver. It can be self-limiting or in some cases, progress to fibrosis (scarring), cirrhosis, or liver cancer. Hepatitis may be caused by toxins, certain drugs, heavy alcohol use, and bacterial and viral infections. Hepatitis viruses are the most common cause of hepatitis in the world [1].

There are 5 main hepatitis viruses, referred to as types A, B, C, D, and E for hepatitis A viruses (HAV), hepatitis B viruses (HBV), hepatitis C viruses (HCV), hepatitis E viruses (HEV) and hepatitis D viruses (HDV) respectively. Each type of hepatitis is caused by a different virus, is spread differently, and accounts for variable prevalence and mortality around the world [1]. Globally, viral hepatitis infection is a major public health problem. Each year, 1.4 million people die from viral hepatitis-related cirrhosis and liver cancer [2]. Prevalence varies according to geographical area. Following Asia, Africa has the second largest number of chronic HBV carriers and is considered to be a region of high endemicity [3]. In 2015, the number of deaths caused by hepatitis viruses resembled the number of deaths caused by tuberculosis and was higher than those caused by HIV [4]. Viral hepatitis mortality has been increasing over time, whereas tuberculosis and HIV mortality has declined. In most cases, mortality due to viral hepatitis in 2015 was caused by chronic liver disease and primary liver cancer, causing some 720,000 and 470,000 deaths respectively [4]. The HCV epidemic affects all regions of the world, with major differences between and within countries [4]. The HDV, a satellite RNA-virus depending on HBV for its propagation. Both HBV and HDV occur worldwide, with an estimated 240 million people worldwide chronically infected with HBV [4,5], of whom approximately 15 to 20 million, or 5% are co-infected with HDV [6]. In combination with HBV, HDV causes the most severe form of viral hepatitis in humans, including fulminant hepatitis and hepatocellular failure, with rapid progression to hepatic cirrhosis followed by hepatic decompensation, and an increased risk of hepatocellular carcinoma [7–9]. Additionally, HBV/HIV coinfection increases the morbidity and mortality beyond the cause of mono-infection by either one of those viruses. The relationship between hepatitis and HIV is important because of the overlapping transmission routes of the two diseases. Hepatitis and HIV can be transmitted via similar contamination routes, such as contact with infected blood, sharing contaminated needles or injection equipment, and drug use. HBV patients coinfected with HIV have higher levels of hepatitis B viremia, have progression to chronic hepatitis B that is approximately five times as fast as that among people infected with only HBV, and have a higher risk of cirrhosis and hepatocellular carcinoma [10].

Although Africa has very high HBV endemicity, HDV prevalence remains poorly known and is thus increasingly studied [4]. Nevertheless, in Africa, of the estimated 65 million chronic HBV carriers, about one-fourth of HBsAg-positive individuals show dual-infection with HDV. Thus, Central Africa has an overall prevalence of 25.6%, West Africa 7.3%, and East and South Africa 0.05% [11].

Numerous seroprevalence studies of viral hepatitis have been conducted in the Central African Republic (CAR) between 1984 and the present day, most focusing on the capital Bangui and its suburbs [12,13]. Although national studies to evaluate national prevalence of HIV (3.5%) [14] and HIV/HBV coinfection have been conducted, in addition to Bangui-centered studies of HBV/HDV [12,15], no studies to date have estimated CAR’s HBV/HDV national prevalence. Previous studies conducted among rural populations in the CAR estimated 2.8% and 10.6% prevalence of hepatitis C virus (HCV) [16] and HBV [17], respectively. This study therefore sought to describe the national geographic distribution of viral hepatitis infections and of HBV/HIV and HBV/HDV co-infections, and to produce a molecular characterization of these viruses among the CAR general population. The seroprevalence of HAV and HEV has not been studied primarily because both viruses are responsible for acute, not chronic infections.

## Methodology

### Study strategy and sample size

**Fig 1:**
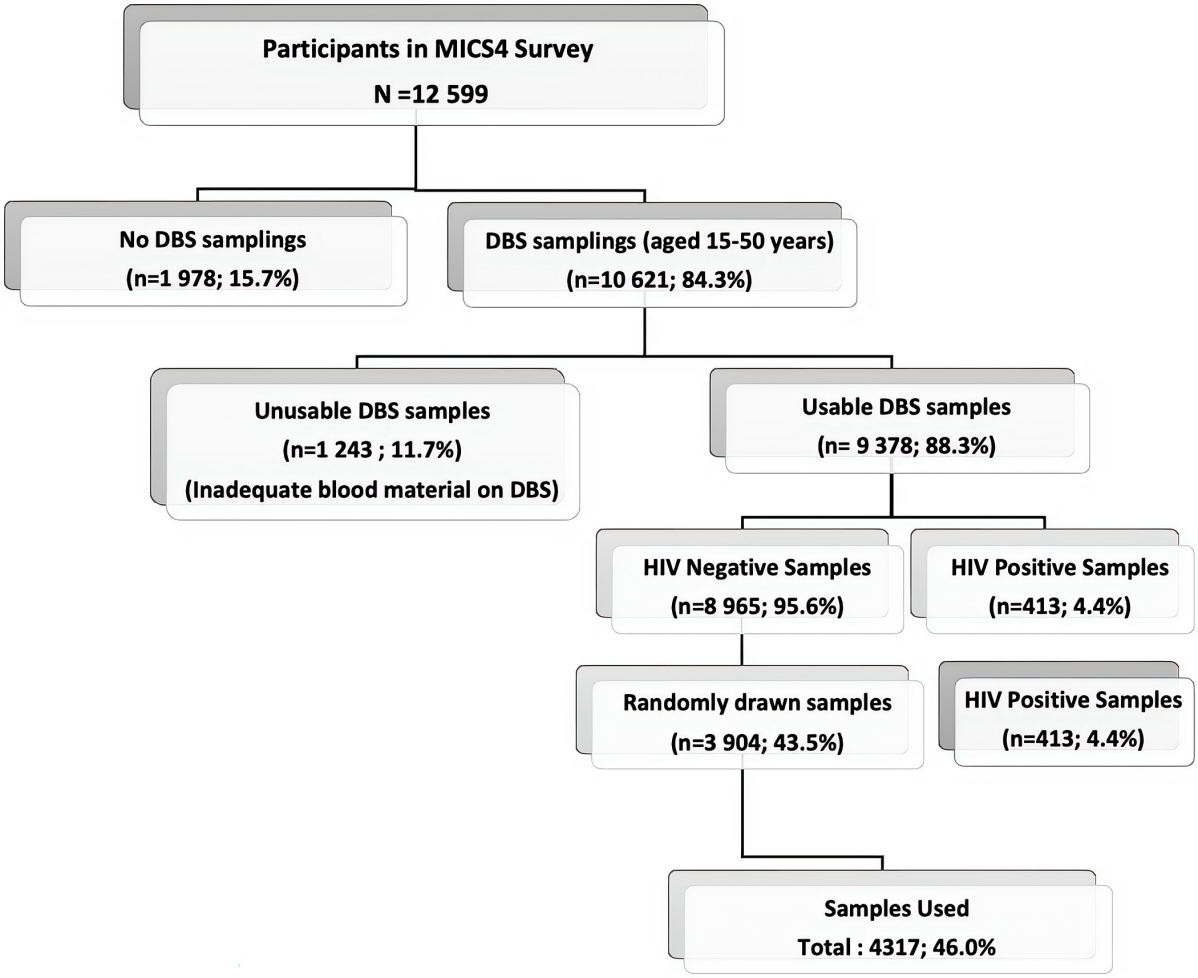
Flowchart of the different stages of randomization of tested samples.

This is a non-interventional descriptive (retrospective) study based on Dried Blood Spot (DBS) samples obtained as part of the 4th Multiple Indicator Cluster Survey (MICS-4) in the CAR in 2010, carried out as part of the United Nations Children’s Fund (UNICEF) program to estimate the prevalence of HIV infection in the country’s general population. We used simple random sampling to give an equal chance for all cases from each region to be included in the sample (Fig 1). Out of the 12,599 persons enrolled during this MICS-4, 10,621 DBS samples were obtained and stored at -20°C.

The sample size for our study was calculated based on the previously reported prevalence of HBsAg (10.6%) [12], a precision of 3% with a 95% confidence interval (CI). From the 10,621 DBS, due to insufficient amounts of some samples, only 9,378 were exploitable, covering all 14 prefectures and Bangui, CAR’s capital city. Samples from two prefectures (Nana-Gribizi and Ouham-Pende) were lost during the storage of DBS. Of the 9,378 exploitable DBS, 413 tested HIV-positive, with 8,965 HIV-negative. Among the 8,965 HIV-negative samples, a random selection was performed to include 3,904 samples in this study, to which we added 413 HIV-positive samples. We used all HIV positive samples to determine HIV/hepatitis co-infection prevalence.

### Socio-demographic data

Socio-demographic data were obtained from the Central African Institute of Statistics, Economic and Social Studies (ICASEES), which collected and archived all data related to each sample, including sex, age, ethnicity, educational level, marital status, residential area (region and urban or rural setting). All data were anonymized before processing the analyses. These data were collected from the standard questionnaire of the survey (www.childinfo.org), adapted to the context of Central African Republic.

### Serological tests

Serological tests for hepatitis B, D, and C viruses were performed using the enzyme-linked immunosorbent assay (ELISA) technique on DBS. Dried blood spot samples were cut and incubated in 200μl Phosphate-buffer saline (PBS) overnight. After vortexing and centrifugation, the supernatant was subjected to ELISA. The HBV markers HBsAg and anti-HBc (total) were tested using Abbott-Murex version 3 (Abbott-Murex Biotech Ltd, Dartford, Kent, UK) for the respective markers. Anti-HDV antibody was tested using the DiaSorin kit for HDV, and Anti-HCV antibody (HCV-Ab) using the Murex anti-HCV kit (DiaSorin, DiaSorin S. P.A. UK Branch Central Road). Serological tests were performed and interpreted according to the manufacturer’s recommendations.

### Nucleic acid extraction and PCR amplification

Viral nucleic acids (DNA/RNA) were extracted with the Qiagen Kits (QIAamp DNA Blood Mini Kit 250 and QIAamp Viral RNA Mini Kit 50) according to the manufacturer’s procedures. Samples were prepared as described for ELISA. Subsequently, the *PreS1* region of the HBV was amplified using a primer pair P1 (5’-TCACCATATTCTTGGGAACAAGA-3’) /P2 (5’-TTCCTGAACTGGGAGCCACCA-3’) with an expected PCR product of 479 bp. For HIV, a nested RT-PCR was used to amplify the protease region of the *pol* gene using two pairs of primers: PRIn1 (5’-TTT-TTT-AGG-GAA-AAT-TTG-3’) /PRIn2 (5’-ATT-TTC-AGG-CCC-AAT-TTT-TGT-3’) and PRIn3 (5’-CAG-ACC-AGA-GCC-AAC-AGC-3’)/PRIn4 (5’-TCT-TCT-GTC-AAT-GGC-CAT-TGT-3) for primary PCR and nested PCR respectively according to the ANRS protocol (http://www.hivfrench resistance.org). The R0 region of HDV was amplified with the primers Casey sens (5’CATGCCGACCCGAAGAGGAAAG3’) and anti-sens (5’GAAGGAAGGCCCTCGAGAACAAGA3’), according to the previously published method [18]. Unfortunately, we did not have enough biological materials to conduct nucleic acid extraction and PCR amplification of HCV; therefore, no HCV strains were sequenced.

### Sequencing

The PCR products were first purified using the Qiagen kit (QIAquick PCR Purification kit, www.qiagen.com/handbooks.), then sent to GATC Biotech (Germany) for sequencing using the Sanger method.

### Sequences analysis

The chromatograms of the sequences obtained were cleaned and analyzed in CLC genomic workbench V.8. The HIV subtypes were determined online, using the Stanford University website (HIV DRUG RESISTANCE DATABASE, http://www.sierra2.stanford.edu). For HBV and HDV, the sequences were aligned and compared with the reference sequences downloaded from the database GenBank (https://www.ncbi.nlm.nih.gov/genbank/), and construction of the phylogenetic trees was carried out using MEGA X software [19].

All sequences obtained were submitted to NCBI and were given the following accession numbers: ***HBV*** *(MT155997- MT156072)*, ***HDV (****MT028414- MT028419)*, *and* ***HIV (****MT121102- MT121107)*. The accession numbers of the reference sequences used in this study are directly mentioned in the phylogenetic threes.

### Statistical analysis

Data were analyzed using STATA Software.14. Percentages with a 95% CI were calculated. HBV-positive and HBV-negative groups were compared using χ2 test or Fisher’s exact test for dichotomous variables and Student’s t-test or Wilcoxon rank-sum test for continuous variables.

All variables associated with HBV in univariate analysis (p < 0.25) were then included in a backward stepwise logistic regression. A *p-value* ≤ 0.05 was considered statistically significant.

### Ethical considerations

Because this study was based partly on retrospective data linked to the fourth Multiple Indicator Cluster Survey (MICS-4) carried out in 2010, which was coupled with HIV screening in the general population, the introduction of a new consent form for participants was neither necessary, recommended or indeed possible.

The protocol anonymised the socio-demographic data stored at the « Institut Centrafricain des Statistiques, des Études Économiques et Sociales » (ICASEES) and participants’ blood samples stored at the Biological Resource Centre of the Institut Pasteur de Bangui. The detection of HIV infection during this survey was based on the anonymous-linked protocol developed by the international DHS (Demographic and Health Surveys) programme and approved by the ICF Macro Ethics Committee. According to this protocol, no name or other individual or geographical characteristic that could identify an individual was linked to a blood sample. The standard MICS-4 questionnaire used (www.childinfo.org) was adapted to the context of the CAR. The “Comité Scientifique chargé de Validation des Protocoles et des Résultats de Recherche en Santé (CSVPRS)” of the Faculty of Health Sciences of the University of Bangui (CAR), which acts as the CAR’s national ethics committee, also approved the specific anonymous-linked protocol for the MICS-4 survey under number 2/UB/FACSS/CSVPRS/10. The tests used to detect HIV infection were strictly anonymous, and it was not possible to inform those tested of the results of their examination. However, at the time of the MICS-4, whether or not they had agreed to be tested for HIV, eligible persons (those providing their consent to be included in the survey) received a coupon to obtain, if they so wished, advice and a free HIV test at a “Centre de Prévention et de Dépistage Volontaire (CPDV)”.

The protocol for this study was also assessed by the CSVPRS, which issued a favorable ethical approval under number 6/UB/FACSS/CSVPRS/12. In the protocol submitted to the CSVPRS, we noted the impossibility of collecting informed consent from participants due to the retrospective nature of the study, which used blood samples stored at the Biological Resources Centre of the Institut Pasteur de Bangui, and the sociodemographic data being anonymous at the outset. We explained that there was no possibility of contacting participants in order to obtain their consent again. The socio-demographic data used to analyse the results of the study were obtained from the ICASEES, which collected and archived all these data in relation to each blood sample. An anonymous number was used to link the biological results to the individual data, but conserved anonymity, again making it impossible to contact participants to obtain their consent.

## Results

### Population characteristics

Among the 4,317 samples analyzed, 2,297 were from males (53.2%) and 2020 from females (46.8%) with a sex ratio of 1.13. The majority of subjects had either primary school or no education (44.8% and 33.4% respectively). More than half of the participants (2356/4317 or 54.6%) identified themselves as of the Protestant religion. The economic quintiles were second (25.0%), poor (22.7%) and medium (22.7%).

### Prevalence of HBV, HCV, HBV/HIV, and HBV/HDV co-infections

Positive HBc-Ab and HBsAg were found in 849 (19.7%) subjects and 555 (12.9%) subjects respectively resulting in a proportion of negative HBsAg/positive HBc-Ab of 294/849 (34.63%). The overall prevalence of anti-HCV-ab was 0.65%. Co-infection of HBV/HIV was found in 49 subjects (1.1%) of the HBV tested population, and HBV/HDV co-infection was found in 92 subjects (16.6%) of the HBV infected population. The prevalence of different hepatitis viral markers and HBV/HIV and HBV/HDV associated with socio-demographic data are reported in *Table 1*.

**Table 1:** Prevalence and distribution of HBsAg, HBc Ab, HCV, HBsAg/HIV and, HBsAg/HDV coinfections.

Characteristics	Number	Percentage	Coinfection HIV/HBsAg			HBsAg			Anti-HBc antibody total			HCV antibody			HDV* antibody		
			Positive (n)	%	*P*	Positive (n)	%	*P*	Positive (n)	%	*P*	Positive (n)	%	*P*	Positive (n)	%	*P*
***Global prevalence***	***4317***	100	49(4317)	1.1		555(4317)	12.9		849(4317)	19.7		28(4317)	0.6		92(555)	16.6	
***Sex***																	
Male	2297	53.2	32(2297)	1.4	0.107	280(2297)	12.2	0.163	443(2297)	19.3	0.503	14(2297)	0.6	0.733	53(280)	18.9	0.133
Female	2020	46.8	17(2020)	0.8		275(2020)	13.6		406(2020)	20.1		14(2020)	0.7		39(275)	14.2	
***Age (years)***																	
15 to 25	1075	24.9	13(1075)	1.2	0.454	147(1075)	13.8	0.696	226(1075)	21.0	0.271	3(1075)	0.3	**0.012**	24(147)	16.3	0.525
26 to 35	1236	28.6	10(1236)	0.8		161(1236)	13.0		254(1236)	20.6		7(1236)	0.6		24(161)	14.9	
36 to 45	961	22.3	10(961)	1.0		122(961)	12.7		177(961)	18.4		4(961)	0.4		18(122)	14.7	
> 45	1045	24.2	16(1045)	1.5		125(1045)	12.0		192(1045)	18.4		14(1045)	1.3		26(125)	20.8	
***School level***																	
No school	1442	33.4	13(1442)	0.9	0.194	184(1442)	12.8	0.822	282(1442)	19.6	0.629	14(1442)	1.0	0.268	32(184)	17.4	0.456
Preschool	2	0.1	0(2)	0.0		0(2)	0.0		0(2)	0.0		0(2)	0.0		0(0)	0.0	
Primary	1934	44.8	20(1934)	1.0		259(1934)	13.4		396(1934)	20.5		12(1934)	0.6		47(259)	2.4	
Secondary and High	848	19.6	13(848)	1.5		101(848)	11.9		155(848)	18.3		2(848)	0.2		12(101)	11.8	
University	91	2.1	3(91)	3.3		11(91)	12.1		16(91)	17.6		0(91)	0.0		1(11)	9.1	
** ***Economic quintile***																	
Poor	979	22.7	10(979)	1.0	0.001	141(979)	14.4	0.155	217(979)	22.2	**0.033**	8(979)	0.8	0.840	25(141)	17.7	0.438
Second	1079	25.0	9(1079)	0.8		134(1079)	12.4		221(1079)	20.5		6(1079)	0.6		20(134)	41.9	
Medium	978	22.6	7(978)	0.7		119(978)	12.2		187(978)	19.1		7(978)	0.7		19(119)	16.0	
Fourth	745	17.3	8(745)	1.1		82(745)	11.0		120(745)	16.1		3(745)	0.4		10(82)	12.2	
Richer	536	12.4	15(536)	2.8		79(536)	14.7		104(536)	19.4		4(536)	0.7		18(79)	22.8	
***Religion***																	
Animist, Buddhist, other	69	1.60	2(69)	2.9	0.053	10(69)	14.5	0.772	15(69)	21.7	0.290	0(69)	0.0	0.842	2(10)	20.0	0.195
Catholic	1344	31.1	8(1344)	0.6		164(1344)	12.2		256(1344)	19.0		9(1344)	0.7		30(164)	18.3	
Muslim	514	11.9	10(514)	1.9		74(514)	14.4		117(514)	22.8		2(514)	0.4		18(74)	24.3	
Atheist	34	0.8	0(34)	0.0		4(34)	11.7		9(34)	26.5		0(34)	0.0		0(4)	0.0	
Protestant	2356	54.6	29(2356)	1.2		303(2356)	12.9		452(2356)	19.2		17(2356)	0.7		42(303)	13.9	

(*): Analysis of HDV in 555 positive AgHBs

(**): The Economic quintile is the economic world bank classification.

**Fig 2:**
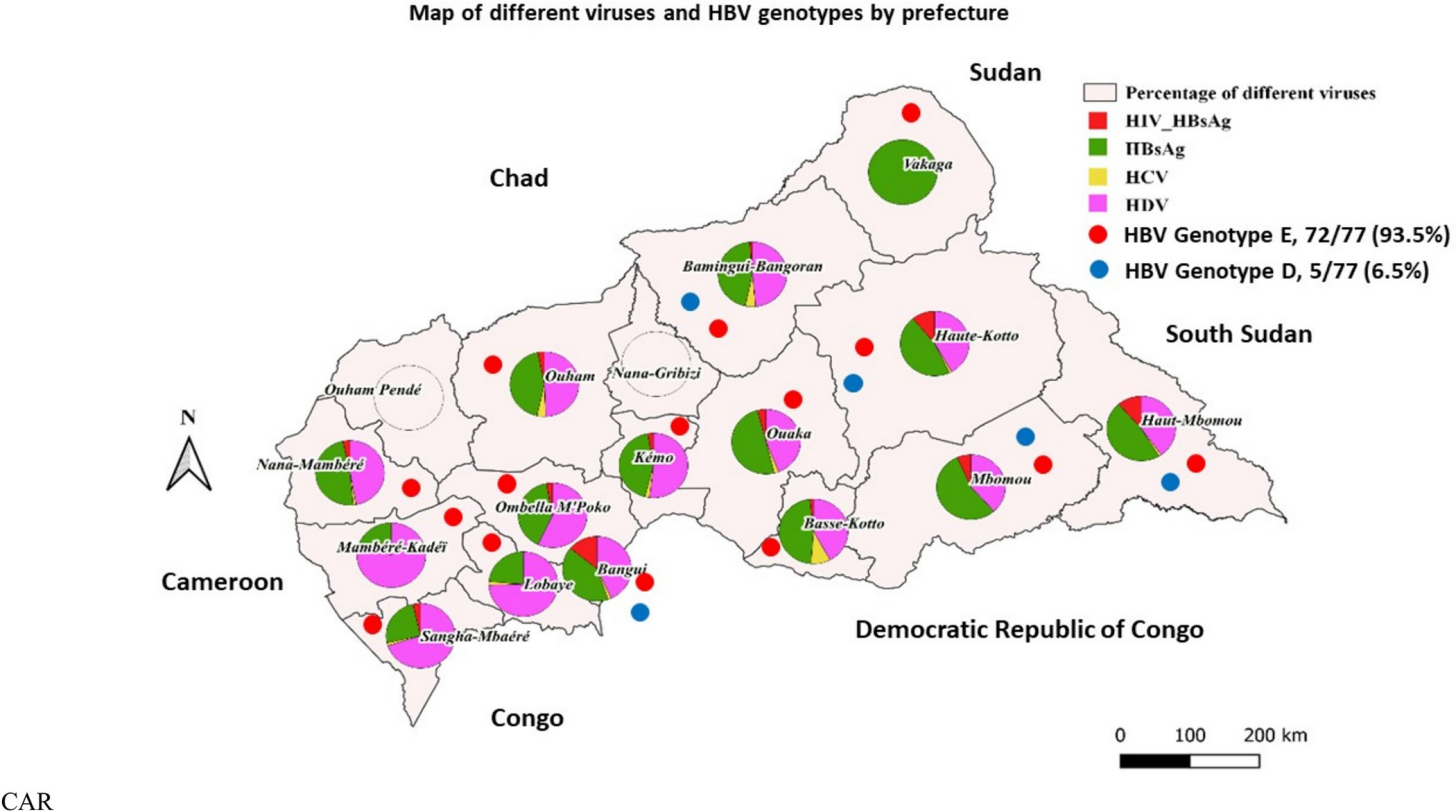
Circulation of viruses and their genotypes in CAR. All four viruses were present in the southeastern part of the Country. Bangui, the capital city, is affected by all three viruses and the circulation of the two genotypes (D and E) of HBV. This map was created with QGIS software, https://www.qgis.org/fr/site/ from the CAF shapefile downloaded from https://data.humdata.org/dataset/cod-ab-caf).

No difference of HBV infection was observed between age groups (p = 0.70), whereas for HCV infection, a significant difference was observed for subjects older than 45 years (p = 0.012). Among districts the prevalence of the four viruses varied (*Table 2 and Fig 2*). Even among districts with the lowest blood samples collection, Vakaga reported the highest prevalence of HBsAg (33.3%), followed by Bamingui-Bangoran (19.5%) and Ouaka (16.9%). However, in Lobaye and Mambere-Kadéi, HBV prevalence was 8.9% and 8.1% respectively. The global prevalence of HBV/HIV co-infection was 1.1%, with the highest prevalence among Bangui samples (5.1%), followed by Haut-Mbomou (3.7%) and Haute-Kotto (3.4%).

**Table 2:** Geographical distribution of hepatitis (B, C and D) viruses.

Geographical distribution	Number	Percentage	Coinfection HIV/HBsAg			HBsAg			HBc Antibody			HCV Antibody			HDV Antibody*		
			Positive (n)	%	*P*	Positive (n)	%	*P*	Positive (n)	%	*P*	Positive (n)	%	*P*	Positive (n)	%	*P*
***Residence/Prefecture***																	
Rural	2704	62.6	20(2704)	0.7	0.001	365(2704)	13.5	0.103	610(2704)	22.6	0.001	13(2704)	0.5	0.075	58(365)	15.9	0.547
Urban	1613	37.4	29(1613)	1.8		190(1613)	11.8		239(1613)	14.8		15(1613)	0.9		34(190)	17.9	
***Prefecture***																	
Bamingui Bangoran	246	5.7	1(246)	0.4	0.001	48(246)	19.5	0.001	103(246)	41.9	0.001	5(246)	2.0	0.031	10(48)	20.8	0.150
Bangui (City of) **	341	7.9	13(341)	3.8		51(341)	15.0		74(341)	21.7		2(341)	0.6		8(51)	15.7	
Basse Kotto	287	6.6	1(287)	0.3		31(287)	10.8		55(287)	19.2		6(287)	2.1		3(31)	9.7	
Haut Mbomou	259	6.0	7(259)	2.7		39(259)	15.1		49(259)	18.9		1(259)	0.4		5(39)	12.8	
Haute Kotto	277	6.4	7(277)	2.5		39(277)	14.1		49(277)	17.7		1(277)	0.4		5(39)	12.8	
Kemo	379	8.8	3(379)	0.8		59(379)	15.6		74(379)	19.5		3(379)	0.8		11(59)	18.6	
Lobaye	281	6.5	0(281)	0.0		25(281)	8.9		28(281)	10.0		2(281)	0.7		7(25)	28.0	
Mambere Kadei	335	7.8	0(335)	0.0		27(335)	8.1		73(335)	21.8		0(335)	0.0		9(27)	33.3	
Mbomou	307	7.1	4(307)	1.3		42(307)	13.7		50(307)	16.3		0(307)	0.0		4(42)	9.5	
Nana Mambere	335	7.8	2(335)	0.6		37(335)	11.0		39(335)	11.6		1(335)	0.3		4(37)	10.8	
Ombella Mpoko	348	8.1	2(348)	0.6		35(348)	10.1		77(348)	22.1		0(348)	0.0		5(35)	14.3	
Ouaka	279	6.5	3(279)	1.1		47(279)	16.8		92(279)	33.0		2(279)	0.7		7(47)	14.9	
Ouham	291	6.7	2(291)	0.7		32(291)	11.0		39(291)	13.4		3(291)	1.0		4(32)	12.5	
Sangha Mbaere	325	7.5	4(325)	1.2		34(325)	10.5		34(325)	10.5		2(325)	0.6		10(34)	29.4	
Vakaga	27	0.6	0(27)	0.0		9(27)	33.3		13(27)	48.1		0(27)	0.0		0(9)	0.0	
***Ethnicity***																	
NIEG(1)	348	8.1	4(348)	1.1	0.739	51(348)	41.1	0.001	82(348)	57.0	0.001	4(348)	1.2	0.626	11(51)	25.6	0.360
Banda	1183	27.4	12(1183)	1.0		179(1183)	15.1		286(1183)	24.2		11(1183)	0.9		31(179)	17.3	
Gbaya	1074	24.9	11(1074)	1.0		110(1074)	10.2		174(1074)	16.2		6)1074)	0.6		20(110)	18.2	
Haoussa	238	5.5	5(238)	2.1		32(238)	13.4		50(238)	21.0		2(238)	0.8		7(32)	21.9	
Mandja	364	8.4	4(364)	1.1		54(364)	14.8		75(364)	20.6		1(364)	0.3		4(54)	7.4	
Mboum	60	1.4	1(60)	1.7		2(60)	3.3		3(60)	5.0		0(60)	0.0		1(2)	50.0	
Ngbaka/Bati	326	7.5	3(326)	0.9		39(326)	12.0		47(326)	14.4		2(326)	0.6		6(39)	15.4	
Sara	149	3.4	0(149)	0.0		16(149)	10.7		29(149)	19.5		0(149)	0.0		2(16)	12.5	
Yakoma/Sango	257	5.9	3(257)	1.2		22(257)	8.6		39(257)	15.2		2(257)	0.8		4(22)	18.2	
Zande/Nzakara	318	7.4	6(318)	1.9		50(318)	1.7		64(318)	20.1		0(318)	0.0		6(50)	12.0	

(*): Analysis of HDV in 555 positive AgHBs.

(**): City of Bangui: Bangui is the capital of Central African Republic.

(1): Non identified ethnic groups.

For ethnic groups, prevalence of HBsAg (41.7%; p< 0.001) and of anti-HBc antibody (57.1%; p< 0.001) was higher among “non identified ethnic groups”, followed by the Banda ethnic group (15.1% and 24.2%), respectively. In contrast, prevalence of HBV/HIV co-infection was slightly higher in the Hausa ethnic group, followed by the Zande/Nzakara ethnic group. Analyses of factors associated with each infection are shown *in Table 3.*

**Table 3:** Association between HBV/HIV coinfection and risk factors.

Caractéristiques	Co-infection HIV/HBV		
	OR	*p*	IC(95%)	
**Economic quintile			
Wealthier	4.25	0.002	[1.71–10.51]
***Residence/Prefecture***			
Urban	2.56	0.001	[1.44–4.56]
***Prefecture***			
Bangui (City of) **	10.14	0.026	[1.31–18.21]
Vakaga					

### Associated risk factors for HIV-HBV co-infection

In our multivariate analysis, being wealthier (OR 4.25, 95% CI: 1.71–10.51) and living in an urban area (OR 2.56, 95% CI: 1.44–4.56), more specifically Bangui and Vakaga (OR 10.14, 95%CI 1.31–78.21), were associated with HIV/HBV co-infection (Table 3).

### Molecular characterization of HBV, HDV, and HIV strains

**Fig 3:**
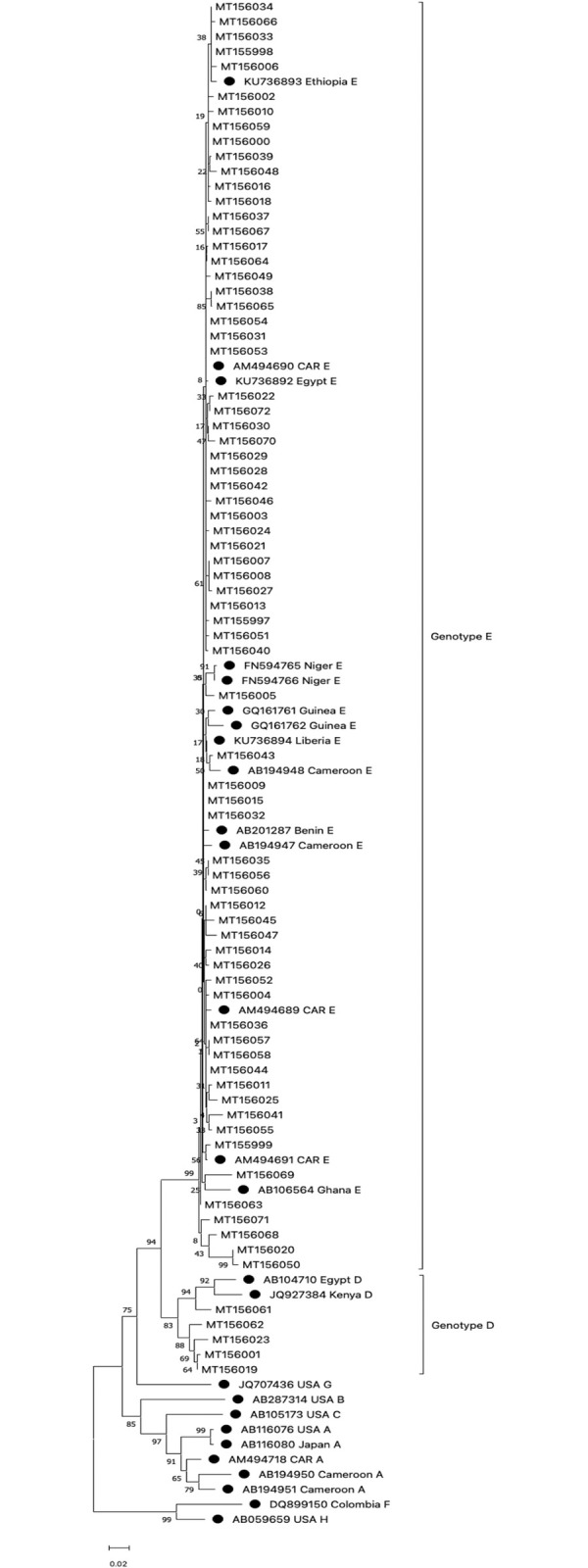
Phylogenetic analyses of 77 HBV strains with their GenBank accession number. The tree was constructed in MEGA using the Neighbor-joining statistical method, the Kimura-2 parameter model, and the bootstrap method of 1000 replicates. The black circles represent reference sequence genotypes A-H downloaded from the database. The remaining are HBV sequences discussed in the present study, and the two genotypes (E and D) are noted on the tree.

**Fig 4:**
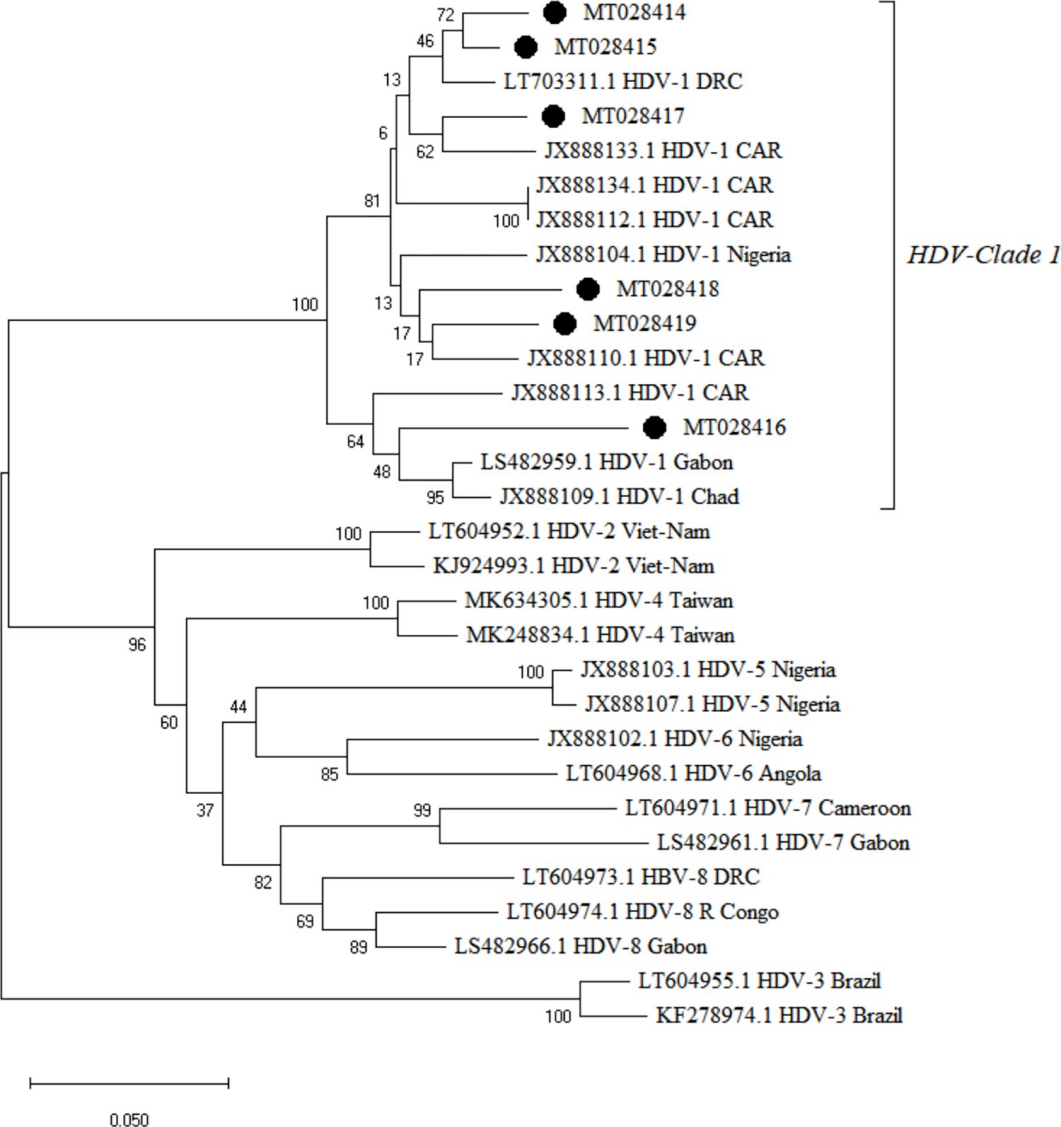
Phylogenetic analyses of the 6 HDV strains. The tree was constructed in MEGA using the Neighbor-joining statistical method, the Kimura-2 parameter model, and the bootstrap method of 1000 replicates. The black circles represent the HDV sequences discussed in the present study, and the remaining are the reference sequences with the country names.

A total of 77 HBV strains based on the *PreS1* region were sequenced. Of these 77 strains, 6 were HBV/HDV co-infected and 6 strains were HBV/HIV co-infected. Of the 77 HBV strains, 72 strains (93.5%) belonged to genotype E, and 5 strains (6.5%) to genotype D (*Fig 3*). Of the 92 HDV positive samples, 6 samples were successfully sequenced and all belonged to clade 1 (*Fig 4*).

Table 4 shows information about the 6 HIV sequences and their GenBank accession numbers. After similarity analysis via the NCBI database and the Stanford University website subtypes-1 (A1 and G), 4 recombinants of subtype-1 were identified.

**Table 4:** Different subtypes of HIV-1 strains.

*Laboratory ID/ GenBank Accession Number*	*Subtypes*	*Similarity (%)*
*CARVIHBG280/ MT121104*	CRF01_AE	100
*CARVIHVK022/ MT121105*	A1	100
*CARVIHSM173/ MT121107*	G	94.6
*CARVIHSM651/ MT121102*	CRF02_AG	100
*CARVIHKE584/ MT121103*	CRF12_BF	100
*CAVIHHK175/ MT121106*	CRF11_cpx	100

## Discussion

The study reported the prevalence of viral hepatitis B, C and Delta biomarkers and the molecular characterization of HBV, HDV, HCV and HIV/Hepatitis coinfection of DBS from a nationwide investigation in the CAR.

In all our analyzed cohorts, HBsAg was highly endemic, but its prevalence varied widely among different regions of the CAR, ranging from 8.1% in Mambere Kadéi to 33.3% in Vakaga. Although it is difficult to compare different age groups, HBsAg prevalence was similar in children and adults: 15 to 25 years (13.7%), 26 to 35 years (13.0%), 36 to 45 years (12.7%), and > 45 years (12.0%). These results resembled those of a previous study showing constancy in HBV prevalence among all age groups in the CAR [20]. This finding may indicate a high burden and an excessive risk of early infection with HBV in children, resulting in a high HDV prevalence (16.6%), which has a similar distribution for HBV across all age groups. Previous studies have designated the CAR as an endemic region for HBV and HDV co-infection [12,15,21,22].

The seroprevalence of HBV and HIV co-infection was 1.1%, demonstrating that the CAR is an endemic region for this co-infection.

In this study, the prevalence of HBc antibody was 19.7%. The distribution of this prevalence follows HBsAg prevalence. The presence of these antibodies indicates that a high proportion of the population had been exposed to HBV. That prevalence is high in rural areas (22.6%; p = 0.001), among the economically poor (22.7%), in the Vakaga and Bamingui Bangoran regions (48.1% and 41.9% respectively; p<0.001), and among other ethnic groups as well as the Banda group (57.1 and 24.2%; p<0.001). Compared to previous studies in CAR, this prevalence has significantly decreased, halving since 2010 when it was 42.3% [23] and declining to 27.1% in rural communities in 2013 [11], to 26.7% among students, and to 21.4% among pregnant women in 2018 [15]. This reduction may be explained by the intensification of hepatitis B surveillance measures for blood transfusions and the introduction of hepatitis B vaccination in the Expanded Program of Immunisation in CAR in 2008.

Although HCV treatment has not been yet initiated in CAR, HCV prevalence in this study was very low (0.6%). Our finding contrasts with a previous review estimating HCV prevalence in Sub-Saharan Africa between 2000 and 2013, which showed West and Central Africa with the highest HCV prevalence [24]. In our study, HCV prevalence in the Bangui samples was 0.6%. We found a higher prevalence in Bamingui-Bangoran samples (2.0%), with variability across regions and no cases at all among the Vakaga samples (Table 2). The Bamingui-Bangoran and Vakaga prefectures are in close proximity, but Vakaga is the most sparsely populated prefecture in the CAR, where very few blood samples were collected compared to other prefectures. We would suggest, then, that socio-demographic and economic factors of these prefectoral populations may account for the differential prevalence of these neighboring regions affecting the prevalence of these viruses.

In addition, the prevalence of anti-HCV was lower in the 15 to 25 age group and higher in those over 45 years old. Similar results have been reported in CAR, Cameroon and Gabon, where massive iatrogenic transmission during medical campaigns through improperly sterilized syringes, needles and the transfusion of contaminated blood products may explain the high prevalence among elderly subjects rather than continuous exposure [25,26].

The main risk factors of HBV identified in our study were economic quintile, religion, residence, prefecture, and ethnicity. The inaccessibility to information tools for prevention, screening, diagnosis, and management of this disease could potentially explain these factors found in this study, but further research is needed to delve deeper into this issue.

From this study, out of the 555 positive HBV samples tested, only 77 were positive in PCR, enabling the sequencing and genotyping of these strains. The reduction in the number of positive PCRs and, consequently, the number of sequenced strains could be attributed to the decreased sensitivity of dried blood spot (DBS) samples caused by storage conditions. This could explain the observed decrease. HBV was categorized into eight genotypes from A to H, based on a divergence of more than 8% in the entire nucleotide sequence of the viral DNA. Two additional genotypes I and J were recently proposed [27]. In the CAR, however, evidence of only three of these HBV genotypes (A, D and E) were reported in previous studies [15,21,28,29].

The present study identified two HBV genotypes (D and E), with HBV genotype E accounting for the dominant strains circulating throughout the CAR (Fig 1). These sequencing results, despite the small sample size, suggest a high degree of concordance with previous studies identifying HBV genotypes E as the most dominant strains circulating in West and Central Africa [21,30]. This HBV genotype has very low diversity and emerged recently, just 200 years or less [30]. HBV genotype D has already been previously described in a Bangui patient with chronic hepatitis [21]. This genotype is predominant in the Maghreb [31], and its low representation in CAR suggests that it may be imported by population and trade movements with other countries in North, West, and Central Africa.

HDV clades (1 to 8) varied across geographic regions [32]. From the present study, all 6 strains belong to clade 1, in accordance with a previous study [33]. The HDV clade 1 has been reported to have rapid and aggressive HDV virion formation and dissemination, making patients infected with this type suffer more adverse outcomes and decreased chances of survival. This finding requires further study of liver disease patients in the CAR.

Among the 6 strains of HIV co-infected with HBV in this study, circulating recombinant forms CRF (CRF01_AE, CRF02_AG, CRF12_BF) and the CRF11_cpx complex were identified. These strains have already been described as part of the pandemic subtypes in Africa [23,33]. In the CAR, the CRF11_cpx complex has been described as predominant. It is associated with antiretroviral resistance, followed by the A1 type [25].

Our study has certain limitations that should be considered, notably the absence of HIV/HCV co-infection, of HBV/HCV/HDV or HIV/HBV/HCV triple infection, and of HIV/HBV/HCV/HDV multiple infection, as well as the absence of detection of HCV-RNA sequences. Our study also had a low proportion of genotyped strains and a lack of molecular confirmation for all serological reactivities. Finally, this study did not include an evaluation of liver function because it relied on a large national demographic survey that did not consider clinical and biological data.

## Conclusion

This study provided a national prevalence and geographical distribution of hepatitis viruses in the CAR, and it has shown that hepatitis viral infections remain a serious public health problem in the country. HBV and HDV remain highly prevalent. HBV prevalence was distributed equally across age groups, and genotype E was dominant. Our results suggested that high prevalence of HBV and HBV/HDV co-infection continue to be responsible for morbidity and mortality, despite the introduction of HBV vaccination in 2008. The data and analysis from this study will be useful in assisting authorities to implement measures to limit HBV, HDV, HCV, and HIV infections and coinfection in the CAR.

## Supporting information

S1 File (ZIP)

S2 File (ZIP)
